# Mitochondrial regulation of cell death: a phylogenetically conserved control

**DOI:** 10.15698/mic2016.03.483

**Published:** 2016-02-23

**Authors:** Lorenzo Galluzzi, Oliver Kepp, Guido Kroemer

**Affiliations:** 1 Equipe 11 labellisée Ligue contre le Cancer, Centre de Recherche des Cordeliers, 75006 Paris, France.; 2 INSERM, U1138, 75006 Paris, France.; 3 Université Paris Descartes/Paris V, Sorbonne Paris Cité, 75006 Paris, France.; 4 Université Pierre et Marie Curie/Paris VI, 75006 Paris.; 5 Gustave Roussy Comprehensive Cancer Institute, 94805 Villejuif, France.; 6 Metabolomics and Cell Biology Platforms, Gustave Roussy Comprehensive Cancer Institute, 94805 Villejuif, France.; 7 Karolinska Institute, Department of Women's and Children's Health, Karolinska University Hospital, 17176 Stockholm, Sweden.; 8 Pôle de Biologie, Hopitâl Européen George Pompidou, AP-HP; 75015 Paris, France.

**Keywords:** autophagy, apoptosis, autosis, ferroptosis, MTP-driven regulated necrosis, necroptosis, parthanatos, pyroptosis

## Abstract

Mitochondria are fundamental for eukaryotic cells as they participate in critical catabolic and anabolic pathways. Moreover, mitochondria play a key role in the signal transduction cascades that precipitate many (but not all) regulated variants of cellular demise. In this short review, we discuss the differential implication of mitochondria in the major forms of regulated cell death.

## INTRODUCTION

Both prokaryotic and eukaryotic cells succumb to very harsh microenvironmental conditions in a virtually instantaneous and uncontrollable manner. Such form of cellular demise, which has been dubbed “accidental cell death” (ACD), reflects the mechanical disassembly of cellular constituents exposed to excessive temperatures, shear forces and/or pressures, and does not involve any molecular machinery 1. In addition, both prokaryotes and eukaryotes have evolved systems that precipitate the death of cells experiencing moderate but unresolvable perturbations of intracellular or extracellular homeostasis 2,3. This latter form of cellular demise, which has been called “regulated cell death” (RCD), relies on the activation of a genetically-encoded machinery, and hence can be modulated by means of pharmacological or genetic interventions 1.

Generally, RCD is activated once adaptive response to stress fail at the cellular level, hence constituting a mechanism for the preservation of organismal homeostasis 4,5,6,7. Defects in the signal transduction cascades that control RCD in eukaryotes have been associated with clinically relevant conditions including acute brain injury, neurodegeneration, cardiac stroke, hepatic damage, and viral infection (all of which are associated with the excessive demise of post-mitotic cells), as well as autoimmune disorders and neoplastic conditions (which are linked to defective RCD) 8,9,10.

Of note, one specific variant of RCD that is known as “programmed cell death” (PCD) is initiated at a predetermined point of a cell’s life, as a part of (post-)embryonic development or the maintenance of tissue homeostasis in the adult 1,11. PCD relies on the same molecular machinery underlying stress-initiated forms of RCD, implying that it can also be retarded or accelerated with specific chemicals or genetic maneuvers 1,11.

The signal transduction cascades controlling RCD have expanded considerably throughout evolution, especially (1) once eukaryotic life has been established (i.e., when organelles including mitochondria became available), and (2) along with the transition from a purely unicellular state to multicellularity (through colonial life) 12,13,14. Nowadays at least five mechanistically distinct variants of RCD have been described in mammals 1,15: (1) intrinsic apoptosis 16,17,18, (2) extrinsic apoptosis 18,19, (3) necroptosis 20,21,22, (4) mitochondrial permeability transition (MPT)-driven regulated necrosis 22,23,24, and (5) ferroptosis 25,26. Moreover, other forms or RCD including parthanatos, autosis and pyroptosis are being characterized with increased precision 27,28,29,30,31. In this short review, we discuss the differential role of mitochondria (which are quintessential for eukaryotic life as they mediate critical bioenergetic and anabolic functions) 32 in the main forms of RCD.

## MITOCHONDRIA AND INTRINSIC APOPTOSIS

Intrinsic apoptosis is a form of RCD initiated by perturbations of intracellular homeostasis that relies on the catalytic activity of the cysteine protease caspase-3 (CASP3) 1,15,16,17,18. In this context, the proteolytic activation of CASP3 is catalyzed by caspase-9 (CASP9), which in turn acquires catalytic activity within a supramolecular complex that is known as “apoptosome” and also contains deoxyATP, the cytosolic adaptor apoptotic peptidase activating factor 1 (APAF1) and an extramitochondrial pool of cytochrome *c*, somatic (CYCS, best known as CYTC) 33,34.

In physiological conditions, CYTC exclusively resides between the outer and the inner mitochondrial membrane, where it is loosely associated with the latter as it operates as an electron shuttle of the respiratory chain 35. Various perturbations of intracellular homeostasis, however, cause the oligomerization of two members of the Bcl-2 protein family, namely BCL2-associated X protein (BAX)- and BCL2-antagonist/killer 1 (BAK1), in the outer mitochondrial membrane, hence altering its permeability to proteins 36. Oligomerized BAX and BAK1 also cause rearrangements of the mitochondrial ultrastructure that facilitate the release of CYTC into the cytosol and hence the activation of the apoptosome 33. Thus, mitochondrial outer membrane permeabilization (MOMP) is a crucial step in the signal transduction cascades that fuel intrinsic apoptosis 36.

In line with this notion, several proteins with prominent anti-apoptotic functions, including various other members of the Bcl-2 family like B-cell CLL/lymphoma 2 (BCL2) itself, BCL2-like 1 (BCL2L1, best known as BCL-X_L_) and myeloid cell leukemia 1 (MCL1), mainly operate by preventing MOMP 37.

There are at least two distinct mechanisms whereby BCL2-like proteins mediate such an effect: (1) by physically interacting with BAX and BAK1 and hence preventing their oligomerization 37; and (2) by sequestering other members of the Bcl-2 protein family that activate BAX and BAK1 in response to stress, the so-called “BH3-only proteins” 38. Moreover, BCL-X_L _has been attributed the capacity to retrotranslocate active BAX to the cytosol (where it normally resides in its inactive state) 39.

Importantly, MOMP drives intrinsic apoptosis not only as it initiates the apoptosome-dependent activation of CASP3 (which cleaves several substrates that are important for cellular survival), but also because it entails the immediate dissipation of the mitochondrial transmembrane potential (Δψ_m_, which is required for ATP synthesis and several other mitochondrial functions) 40,41. This implies that intrinsic apoptosis can occur even in the absence of APAF1, CASP9 and CASP3 (or in the presence of chemical agents specifically targeting these proteins) 1. However, the inhibition of APAF1, CASP9 or CASP3 generally delays intrinsic apoptosis and alters several of its manifestations 1. Indeed, CASP3 is mechanistically responsible for various biochemical, morphological and immunological features of apoptosis, including the exposure of phosphatidylserine (PS) on the surface of dying cells 42,43, DNA fragmentation (which underlies nuclear condensation) 44,45, and the release of the immunosuppressive factor prostaglandin E_2_ (PGE_2_) 46. In spite of the precise kinetics of the process, mitochondria play a key role in the signal transduction cascades that precipitate intrinsic apoptosis.

## MITOCHONDRIA AND EXTRINSIC APOPTOSIS

Extrinsic apoptosis is a CASP3-dependent form of RCD initiated by perturbations of the extracellular microenvironment 1,15,19,47. Extrinsic apoptosis can be elicited by two classes of plasma membrane receptors that operate in a diametrically opposed fashion: (1) so-called “dependence receptors”, which acquire pro-apoptotic activity when the concentration of their ligands falls below a specific threshold 47; and (2) so-called “death receptors”, which trigger RCD in the presence of their ligands 19. The molecular mechanisms bridging dependence receptors to the transmission of an RCD-promoting signal have not been elucidated yet, and appear to exhibit a remarkable degree of context-dependency 47. Thus, while unbound patched 1 (PTCH1) and deleted in colorectal carcinoma (DCC) appear to interact with the cytosolic adaptor four and a half LIM domains 2 (FHL2, best known as DRAL) to assemble a supramolecular complex that promotes the activation of CASP9 48,49, other dependence receptors like unc-5 netrin receptor B (UNC5B) have been shown to respond to ligand withdrawal by triggering a death-associated protein kinase 1 (DAPK1)-dependent signaling pathway 50.

The signal transduction cascades activated by death receptors upon ligand binding, conversely, are well characterized. Normally, FAS trimers (which assemble and disassemble spontaneously) get stabilized in the presence of FAS ligand (FASLG), favoring the recruitment of a large multiprotein complex at the cytosolic tail of the receptor 19. This supramolecular entity, which is known as “death-inducing signaling complex” (DISC), contains receptor-interacting protein kinase 1 (RIPK1), FAS-associated protein with a death domain (FADD), various isoforms of CASP8 and FADD like apoptosis regulator (CFLAR, best known as c-FLIP) as well as several members of the baculoviral IAP repeat containing (BIRC) protein family (which act as E3 ubiquitin ligases), and operates as an activating platform for caspase-8 (CASP8) or caspase-10 (CASP10). CASP8 (as well as CASP10) can catalyze the proteolytic activation of CASP3, hence precipitating apoptotic RCD 51,52, while the other components of the DISC either (1) play structural roles (like FADD does), (2) mediate direct RCD-inhibitory functions (like BIRC proteins and c-FLIP do), or (3) connect DISC activation to other signal transduction cascades including the activation of the pro-inflammatory transcription factor NF-κB (like RIPK1 does) 53.

Importantly, distinct death receptors assemble structurally different DISCs upon activation, implying that the signaling pathway initiated by death receptors can exhibit a remarkable degree of variation (although they generally culminate in CASP8 or CASP10 activation) 53. In some cell types (which are commonly referred to as Type I cells, e.g., lymphocytes), the activation of CASP8 by the DISC is perfectly sufficient to drive CASP3-dependent apoptotic RCD 54. However, in other cell types (which are commonly indicated as Type II cells, e.g., hepatocytes), the optimal activation of CASP3 by CASP8 critically relies on MOMP 54. In this setting, MOMP is driven by the CASP8-catalyzed activation of BH3 interacting domain death agonist (BID), a potent BH3 only protein 55,56. Whether cells behave in a Type I or Type II manner upon death receptor ligation depends on the cytosolic abundance of X-linked inhibitor of apoptosis (XIAP), a BIRC family members that exerts potent caspase-inhibitory functions 57. Thus, mitochondria play an active role in some (but not all) instances of extrinsic apoptosis.

## MITOCHONDRIA AND NECROPTOSIS

Necroptosis is a variant of RCD that obligatorily relies on the activation of the RIPK1-like protein receptor-interacting protein kinase 3 (RIPK3) and the pseudokinase mixed lineage kinase domain-like (MLKL), and generally manifests with a necrotic morphology 1,15,20,21,22. Various (but not all) instances of necroptosis also impinge on the activation of RIPK1 itself, implying that they can be retarded by the RIPK1-targeting agent necrostatin-1 (Nec-1). For instance, this applies to necroptosis elicited by tumor necrosis factor receptor superfamily member 1A (TNFRSF1A) ligation in CASP8-deficient conditions 58,59,60. Heterotrimeric complexes containing CASP8, FADD and the long isoform of c-FLIP operate indeed as tonic inhibitors of necroptosis, normally preventing the activation of this RCD modality upon death receptor ligation 61,62. However, when RIPK1 ubiquitination by BIRC family members is chemically antagonized (with agents commonly known as Smac mimetics) and CASP8 is absent or blocked, prolonged TNFRSF1A signaling efficiently drive the assembly of a RIPK1- and RIPK3-containing complex that phosphorylates MLKL, endowing it with the ability to translocate to the inner leaflet of the plasma membrane and compromise its structural integrity 63,64,65,66.

Initially, mitochondria were thought to participate in necroptotic signaling in at least two ways: (1) necroptosis was linked to an oxidative burst caused by the RIPK3-dependent activation of various metabolic enzymes, including mitochondrial glutamate dehydrogenase 1 (GLUD1) 67, and (2) MLKL was suggested to boost the catalytic activity of PGAM family member 5, serine/threonine protein phosphatase, mitochondrial (PGAM5), resulting in the activating dephosphorylation of dynamin 1-like (DNM1L, best known as DRP1) and consequent mitochondria fragmentation 68,69. Subsequent evidence from several independent laboratories, however, demonstrated that mitochondria are completely dispensable for necroptosis. Indeed, necroptotic signaling was found to be normal in cells lacking mitochondria upon a widespread mitophagic response 70, as well as in cells from *Pgam5^-/-^* mice 71. Very recent findings linking MLKL to mitochondrial MCL1 depletion and consequent MOMP remain to be verified 72. Thus, necroptosis should be considered as a mitochondrion-independent form of RCD.

## MITOCHONDRIA AND MPT-DRIVEN REGULATED NECROSIS

The term MPT is commonly employed to indicate an abrupt increase in the permeability of the inner mitochondrial membrane to small solutes, resulting in immediate Δψ_m_ dissipation, massive water intake, and osmotic organelle breakdown 1,15,22,23,24. According to current models, the MPT ensues a conformational change in a multiprotein complex assembled at the juxtaposition between the inner and outer mitochondrial membranes, the so-called “permeability transition pore complex”, (PTPC) 17,24. The precise molecular composition of the PTPC remains matter of debate and may exhibit considerable degree of context dependency 17,24. However, at least one protein has been attributed a key, non-redundant role in MPT, i.e., peptidylprolyl isomerase F (PPIF, best known as CYPD) 73,74,75. Recent findings suggest that also the c subunit of the F_O_ ATPase (which in humans exists in 3 isoforms, ATP5G1-3) plays a critical function within the PTPC 76, yet compelling genetic evidence in support of this hypothesis is difficult to obtain. Irrespective of this unknown, MPT results in a rapid drop of intracellular ATP availability, driving a form of RCD that generally manifests with necrotic morphological features 17,44. As per definition, MTP-driven regulated necrosis occurs with a delayed kinetics in cells lacking CYPD, as well as in the presence of the chemical CYPD inhibitor cyclosporin A (CsA) 1,15. Thus, mitochondria play a fundamental role in the signal transduction cascades underlying MPT-driven regulated necrosis.

## MITOCHONDRIA AND FERROPTOSIS

Ferroptosis is an iron-dependent form RCD generally initiated by the inhibition of plasma membrane system x_C_- (a cystine/glutamate antiporter), resulting in the depletion of antioxidant defenses and lethal lipid peroxidation 1,15,25,26. Ferroptosis is under the endogenous control of cytosolic glutathione peroxidase 4 (GPX4) 77,78, and can be delayed by the small molecule ferrostatin-1 (Fer-1) as well as by other chemical agents that inhibit lipid peroxidation 79.

Of note, Fer-1 and alike fail to inhibit the generation of mitochondrial reactive oxygen species (ROS) 79. Moreover, ferroptosis proceeds normally in *Ppif^-/-^* cells as well as in the presence of the MPT inhibitor CsA 80. Thus, it seems that mitochondria and mitochondrial ROS are perfectly dispensable for ferroptosis, although this conjecture has not yet been addressed experimentally in a direct fashion.

## MITOCHONDRIA AND OTHER FORMS OF RCD

### Parthanatos

Parthanatos is a peculiar form or RCD depending on poly(ADP-ribose) polymerase 1 (PARP1), a nuclear protein involved in DNA repair, and apoptosis inducing factor, mitochondria associated 1 (AIFM1) 1,15,81. PARP1 hyperactivation by DNA alkylating agents entails a very pronounced depletion in intracellular NAD^+^ stores, resulting in a potentially lethal bioenergetic crisis 82. Moreover, poly(ADP-ribose) moieties generated by PARP1 appear to bind AIFM1 in the mitochondrial intermembrane space, hence favoring its release to the cytosol 83. Upon binding to peptidylprolyl isomerase A (PPIFA, best known as CYPA), extramitochondrial AIFM1 acquires the ability to translocate to the nucleus and mediate large-scale DNA fragmentation 83. Mitochondria are therefore required for parthanatos to proceed according to a normal kinetics.

### Autosis

Autosis is a variant of autophagic cell death, i.e., a form of RCD that is precipitated by the molecular machinery for macroautophagy 1,15,27,28. In addition, autosis impinges on the plasma membrane Na^+^/K^+ ^ATPase, implying that it can be modulated with chemical agents that target this ionic pump, like cardiac glycosides 27,84. The morphological manifestations of autosis differ from those of classical apoptosis and necrosis, encompassing a pathognomonic dilation of the perinuclear space and the massive accumulation of autophagic vacuoles in the cytoplasm 27,28,44. Although some components of the molecular machinery for macroautophagy interact with mitochondrial proteins (including BCL2), the involvement of mitochondria in the signal transduction cascades that precipitate autosis has not been investigated yet.

### Pyroptosis

Pyroptosis is a form of RCD that critically rely on the cleavage of gasdermin D (GSDMD) by inflammatory caspases, i.e., caspase-1 (CASP1), caspase-4 (CASP4), caspase-5 (CASP5) or caspase-11 (Casp11, the mouse orthologue of human CASP4 and CASP5) 1,15,29,30,31. Thus, pyroptosis is generally associated with the assembly and activation of so-called “inflammasomes”, which are supramolecular platforms that promote the CASP1-, CASP4-, CASP5- or Casp11-dependent proteolytic processing of pro-interleukin-1β (pro-IL-1β) and pro-interleukin-18 (pro-IL-18) 85. These observations imply that pyroptosis (1) can only occur in cell types that express sufficient amount of inflammatory caspases (e.g., cells of the monocytic lineage) 86, (2) is associated with the release of mature IL-1β and IL-18 86, and (3) is sensitive to broad-spectrum caspase inhibitors like Z-VAD-fmk (which also delays apoptosis) as well as to chemicals that specifically block CASP1, CASP4, CASP5 or Casp11 (which have no effects on apoptosis) 1.

Morphologically, pyroptosis manifests with features that resemble (at least in part) those of apoptosis 44,87. Importantly, mitochondrial ROS have been shown to act as intracellular danger signals and promote inflammasome activation coupled CASP1-dependent RCD in some cells 88. However, the integrity of mitochondria appears to be preserved in the first phases of pyroptotic signaling 89,90,91.

In summary, it remains to be formally demonstrated whether mitochondria are a core component of the signal transduction cascades that precipitate pyroptosis or whether they simply act as pyroptosis initiators in specific pathophysiological settings.

## CONCLUDING REMARKS

**Figure 1:**
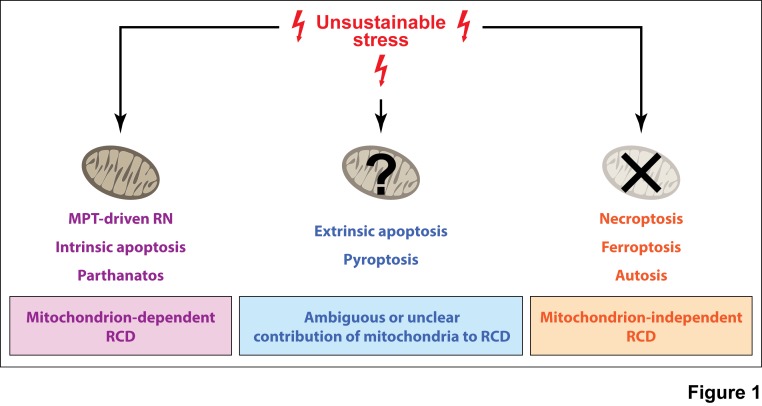
FIGURE 1: Implication of mitochondria in RCD signaling. Mitochondria play a key, non-redundant role in the signal transduction cascades that precipitate intrinsic apoptosis, some instances of extrinsic apoptosis, mitochondrial permeability transition (MPT)-driven regulated necrosis (RN), and parthanatos, but are completely dispensable for necroptosis, ferroptosis, and autophagic cell death by autosis (at least according to current knowledge). The actual contribution of mitochondria to the signaling pathways that drive pyroptotic regulated cell death (RCD) remains to be formally elucidated.

The signal transduction cascades that precipitate RCD have become increasingly more complex with evolution, especially along with the acquisition of the eukaryotic state and multicellularity 12,13,14,92. Modern prokaryotes harness RCD to favor the survival of the species when colonies are threatened by environmental conditions 93,94, and it seems that such an evolutionarily ancient capacity has been fixed by evolution. Mitochondria (the remnants of bacteria that at some stage were incorporated into protoeukaryotes to generate eukaryotic life) play indeed a fundamental function in some (but not all) RCD-stimulating pathways in modern eukaryotes (**Figure 1**). Interestingly enough, evolutionarily ancient eukaryotes including *Saccharomyces cerevisiae* mostly (if not exclusively) rely on mitochondrion-dependent forms of RCD 92,95,96,97. Conversely, mitochondrion-dependent RCD variants seem to have completely disappeared in post-mitotic animals like *Caenorhabditis elegans* 98 and *Drosophila melanogaster* 99. Taken together, these observations suggest that mitochondrion-dependent variants of RCD may have evolved before their mitochondrion-independent counterparts.

In conclusion, mitochondria are quintessential for eukaryotic cells, not only as they mediate fundamental bioenergetic and anabolic functions, but also as they contribute to several (but not all) signal transduction cascades that precipitate RCD.
